# Mesoscopic-scale grain formation in HfO_2_-based ferroelectric thin films and its impact on electrical characteristics

**DOI:** 10.1186/s40580-022-00342-6

**Published:** 2022-11-12

**Authors:** Masaharu Kobayashi, Jixuan Wu, Yoshiki Sawabe, Saraya Takuya, Toshiro Hiramoto

**Affiliations:** 1 d.lab, School of Engineering, The University of Tokyo, 4-6-1 Komaba Meguro-Ku, Tokyo, 153-8505 Japan; 2 Institute of Industrial Science, The University of Tokyo, 4-6-1, Komaba Meguro-Ku, Tokyo, 153-8505 Japan

**Keywords:** Ferroelectric HfO_2_, Thermodynamics, Kinetics

## Abstract

Ferroelectric memory devices are expected for low-power and high-speed memory applications. HfO_2_-based ferroelectric is attracting attention for its CMOS-compatibility and high scalability. Mesoscopic-scale grains, of which size is almost comparable to device size, are formed in HfO_2_-based ferroelectric poly-crystalline thin films, which largely influences electrical characteristics in memory devices. It is important to study the impact of mesoscopic-scale grain formation on the electrical characteristics. In this work, first, we have studied the thickness dependence of the polarization switching kinetics in HfO_2_-based ferroelectric. While static low-frequency polarization is comparable for different thickness, dynamic polarization switching speed is slower in thin Hf_0.5_Zr_0.5_O_2_ (HZO) capacitors. Based on the analysis using the NLS model and physical characterization, thinner HZO contains smaller grains with orientation non-uniformity and more grain boundaries than thicker HZO, which can impede macroscopic polarization switching. We have also theoretically and experimentally studied the polar-axis alignment of a HfO_2_-based ferroelectric thin film. While in-plane polar orientation is stable in as-grown HZO, out-of-plane polarization can be dominant by applying electric field, which indicates the transition from in-plane polar to out-of-plane polar orientation in the ferroelectric phase grains. This is confirmed by calculating kinetic pathway using ab-initio calculation.

## Introduction

**Fig. 1:**
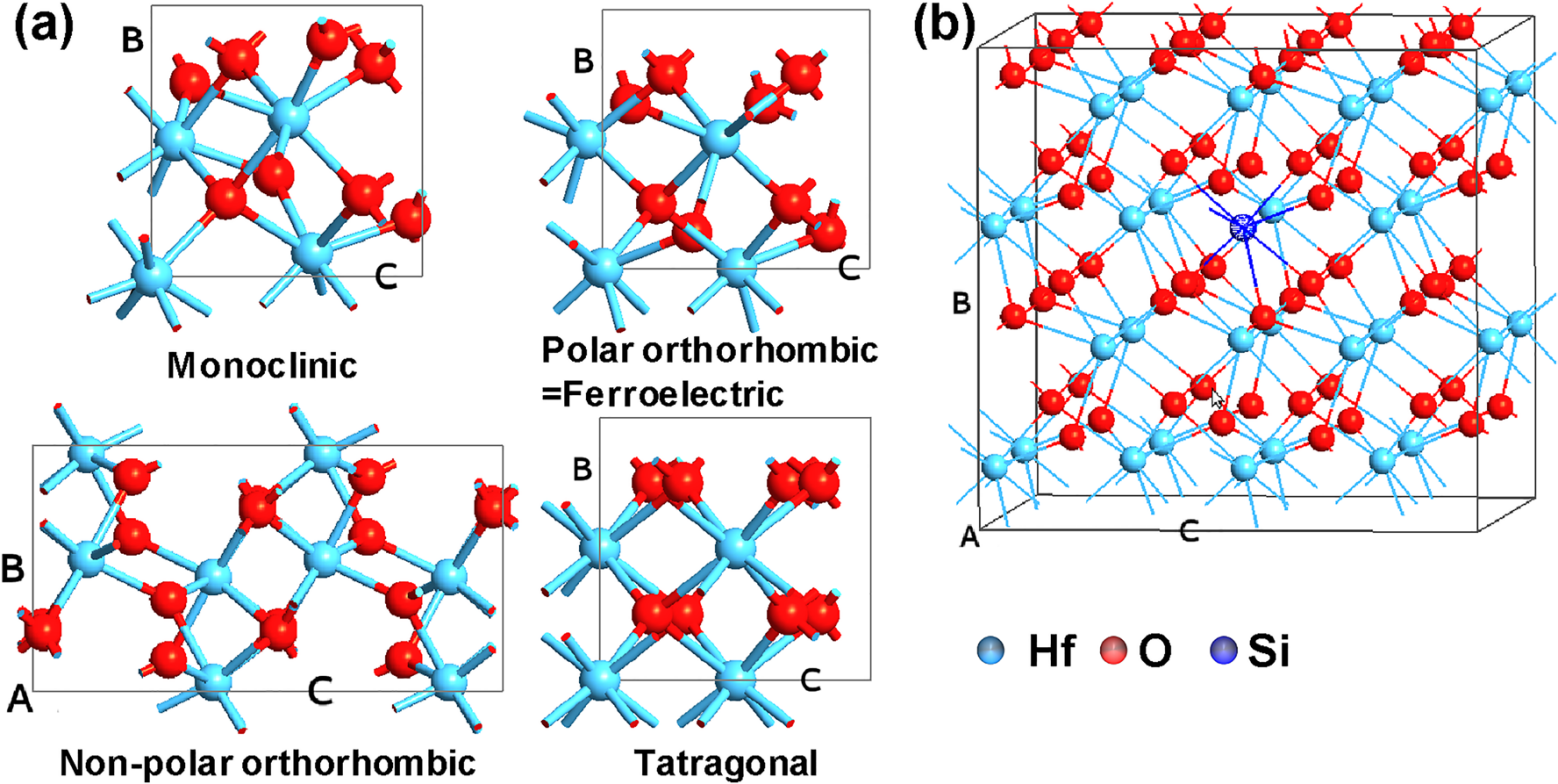
(**a**) Unit cell structures of the monoclinic (m), polar orthorhombic (ferroelectric, f), non-polar orthorhombic (o), and tetragonal (t) phases, (**b**) Supercell structure with dopants, which is expanded by 2 × 2 × 2 for monoclinic, tetragonal and ferroelectric phases and by 2 × 2 × 1 for non-polar orthorhombic phase

Ferroelectric memory devices are promising candidates for low-power and high-speed memory for IoT and AI applications. Since ferroelectricity was discovered in HfO_2_-based material, high-capacity and high-density are also expected in ferroelectric memory devices [1, 2]. In fact, there have been manufacturing-level demonstrations of scaled ferroelectric memory devices, including 1-transistor-1-Capacitor FeRAM [3, 4], 1-transistor FeFET [5, 6] and 1-resistance FTJ devices [7] using HfO_2_-based ferroelectric. For development toward high-volume manufacturing, understanding and engineering the material property is indispensable to realized high-performance and high-reliability ferroelectric memory devices. Till now, the physical mechanism of the emergence of ferroelectricity in HfO_2_-based ferroelectric has been intensively studied [8–11]. Ferroelectricity in HfO_2_-based material comes from the polar orthorhombic Pca2_1_ phase (ferroelectric phase, f-phase) (Fig. 1). Bulk-HfO_2_ has the stable phases of the monoclinic phase (m-phase) around room temperature and the tetragonal phase (t-phase) at high temperature, both of which are centrosymmetric without showing ferroelectricity. It has been reported that film thickness, strain, dopant and electric field facilitate the f-phase formation [12–16].

**Fig. 2:**
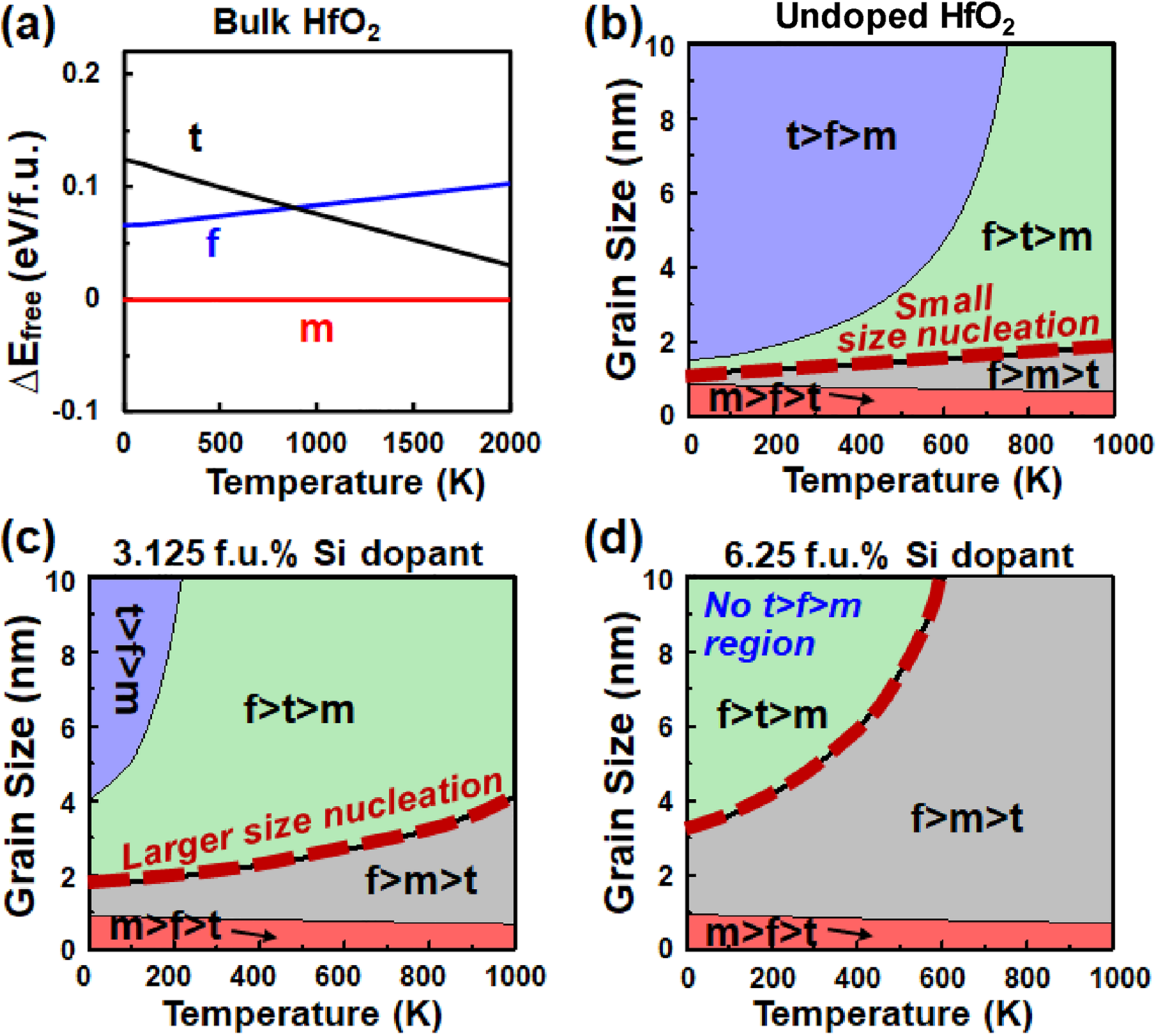
(**a**) Calculated free energy of bulk HfO_2_ at finite temperature with reference to the m-phase. Phase order diagrams based on free energies of m, t, f-phases of (**b**) undoped HfO_2_, (**c**) 3.125 f.u. % Si doped HfO_2_, and (**d**) 6.25 f.u. % Si doped HfO_2_

**Fig. 3:**
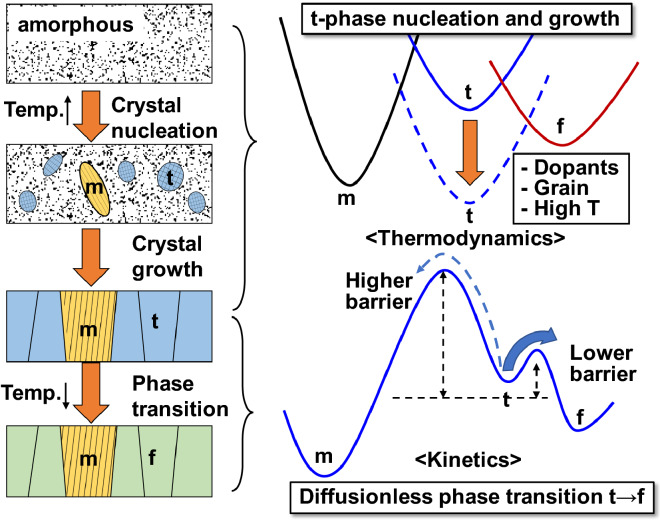
Schematic illustration of the mechanism of the f-phase formation from amorphous HfO_2_ in thermal process

As a case study, essential factors of dopant, surface energy, and temperature are considered, and reviewed here [17]. The role of dopant such as Si and Al is the lowering of total energy in the t-phase in bulk-HfO_2_. When it comes to a thin film, the surface energy further stabilizes the t-phase and the t-phase becomes the most stable state against the m-phase with a finite grain size. High temperature is applied during thermal annealing process. Figure 2a shows the bulk free energy of HfO_2_ versus temperature. Figure 2b–d show phase diagrams as a function of temperature and grain size based on the free energies of undoped HfO_2_ and doped HfO_2_ grains. As the t-phase has higher entropy than other phases, the t-phase is further stabilized in larger grain size at high temperature. Thus, in a thin film, t-phase grains nucleate and grow from an amorphous film. Note that a too thick film allows large grain growth of the m-phase, which should be avoided. In cooling down, the phase transition occurs from the t-phase to the f-phase. This is because the f-phase is a metastable phase with lower energy than the t-phase, and kinetic energy barrier height from the t-phase to the f-phase is lower than to the m-phase. Note that a too thin film does not allow the phase transition from the t-phase to f-phase because the f-phase is not a metastable phase with very small grains. The overall ferroelectric phase formation is summarized in Fig. 3 which illustrates the thermodynamics and kinetics of the phase formation from amorphous to the f-phase through the thermal process.

Unlike perovskite ferroelectric material, HfO_2_-based ferroelectric is grown on metal electrode materials such as TiN, TaN, and W which are commonly used in CMOS interconnect. Thereby, HfO_2_-based ferroelectric is formed as a poly-crystalline film. The grain size is in mesoscopic scale; nanometer size and comparable to device size. The grain size and characteristics variability are concerns for manufacturing and actively discussed [18, 19]. Particularly, we are interested in how grains are distributed in thin HfO_2_-based ferroelectric film and influence static and dynamic characteristics. For practical application, thinner HfO_2_-based ferroelectric films are preferred as operation voltage can be reduced. Thus, it is important to systematically study the thickness dependence of the relationship between the physical property and electrical characteristics of poly-crystalline HfO_2_-based ferroelectric thin film. We are also interested in how grains are oriented in thin HfO_2_-based ferroelectric film. To realize 3D-structure ferroelectric device, the polar axis should be aligned perpendicular to the surface [20]. It is important to study preferable orientation and figure out how to control the orientation alignment.

Based on the above motivations, in this paper, we report the thickness dependence of the polarization switching kinetics and the polar-axis alignment in HfO_2_-based ferroelectric poly-crystalline thin film, considering the mesoscopic-scale grain formation.

## Methods/experimental

Metal/ferroelectric/metal (MFM) capacitors were fabricated by following the process below. A 30 nm-thick TiN layer was deposited on a wet-cleaned N^+^-Si substrate as a bottom electrode by RF sputtering at room temperature. Next, Hf_0.5_Zr_0.5_O_2_ (HZO) layers were deposited by ALD at 250 °C with thicknesses of 6.5 nm, 8.4 nm, and 12.0 nm. Then another 30 nm-thick TiN layer was deposited as a top electrode by RF sputtering at room temperature. Lastly, RTA was done at 500 °C in N_2_ ambient for 10 s duration. Nominal capacitor size is 15 µm. Static polarization-voltage (*P*–*V*) curves were obtained by low-frequency measurement with 1 kHz triangular voltage pulses and a charge amplifier circuit. Dynamic switched polarization charges were obtained by applying initialization set/reset pulses, write voltage pulse and then read double voltage pulses. Write voltage pulse width (*t*_pw_) and amplitude (*E*_FE_) were varied. We define write operation by positive voltage as program and write operation by negative voltage as erase. Thereby, the samples are consistently initialized, and only switched polarization charge can be extracted by subtracting the non-switched charge at the second read pulse from the total charge at the first read pulse. Note that our measurement system limits the minimum pulse width to 50 ns due to the band width. We confirmed that switched polarization charge does not strongly depend on the capacitor pad size down to 100 ns. Our measurement ensures the accuracy in the medium speed region. In the physical characterization, TEM and electron diffraction mapping were used for both cross-sectional and plan-view images. The top TiN electrode was stripped by wet H_2_O_2_ solution without causing damage before analysis. The electron diffraction mapping method provides the distribution of grain phases and crystal orientations [21]. Ab-initio simulations were employed for supporting the discussion, the detail of which is described in our previous report [17]. The default setting is as follows: The exchange–correlation energy is computed using a generalized gradient approximation-Perdew-Burke-Ernzerhof (GGA-PBE). The SG15 Optimized Norm-Conserving Vanderbilt pseudopotential (ONCV) is used for all calculations. The density mesh cutoff is 140 Har. For unit-cell calculations, optimization is carried out for both lattice and atomic position relaxation with a force tolerance of 0.01 eV/Å. The Brillouin zone is sampled with 5 × 5 × 5 k-points. The surface energy is calculated by a slab model with different orientations. The slab is built with a 6 unit-cell HfO_2_ in the out-of-plane direction and 3 unit-cell vacuum at both ends. In-plane directions are periodic with 1 unit-cell.

## Results and discussion

### The thickness dependence of polarization switching kinetics and grain distribution

**Fig. 4:**
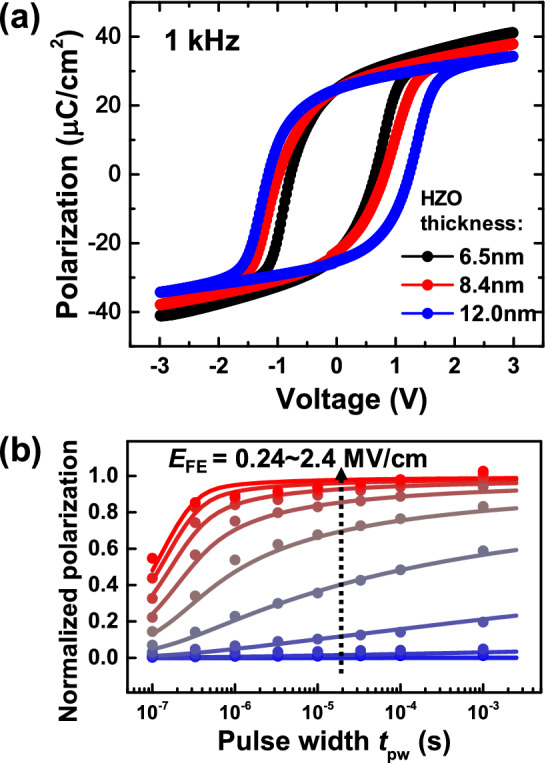
(**a**) Measured *P*–*V* curves of the HZO capacitors with HZO thicknesses of 6.5 nm, 8.4 nm, and 12.0 nm at 1 kHz, (**b**) measured normalized polarization curves versus pulse width *t*_pw_ as a function of applied electric field *E*_FE_. for the 8.4 nm-thick HZO capacitor

**Fig. 5:**
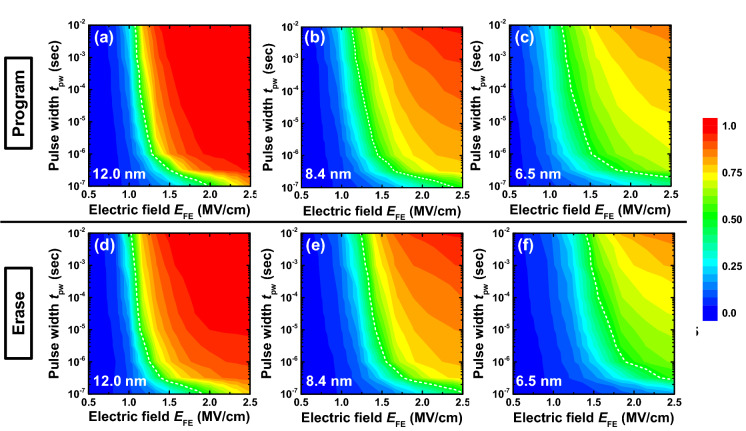
Measured contour plots of normalized switching polarizations as a function of *t*_pw_ and *E*_FE_ for program operation with (**a**) 12.0 nm, (**b**) 8.4 nm, and (**c**) 6.5 nm-thick HZO capacitors, and for erase operation with (**d**) 12.0 nm, (**e**) 8.4 nm, and (**f**) 6.5 nm-thick HZO capacitors

Low-frequency *P*–*V* curves were measured with different thicknesses as shown in Fig. 4a. Clear ferroelectricity was observed. Remanent polarization (2*P*_r_) was almost the same for all the thicknesses, which means that the static characteristics was almost the same for all thicknesses in terms of ferroelectric polarization charge. Coercive field (2*E*_c_) was estimated to be 2.2, 2.1, and 2.0 MV/cm for 6.5, 8.4, and 12.0 nm, respectively. 2*E*_c_ is slightly larger in the thinner film but is not largely different. Next, switched polarization was measured as a function of *t*_pw_ and *E*_EF_ by the input waveform as described in Ref. 23. Switched polarization is normalized by the saturated polarization value. Figure 4b shows the example of measured switched polarization curves for the 8.4 nm-thick HZO capacitor. To compare switching speed of the ferroelectric capacitors with different thicknesses, switched polarization curves can be plotted in the form of a contour plot. Figure 5 show contour plots of the normalized switched polarization as a function of *t*_pw_ and *E*_EF_ for each thickness and for both program and erase operation. Dotted lines represent the conditions of *t*_pw_ and *E*_FE_ where half of the total polarization charge is switched. For both program and erase operation, switching speed becomes slower for the thinner HZO capacitor at each *E*_FE_, and electric field required for switching becomes larger for the thinner HZO capacitor at each *t*_pw_. Erase operation is slightly slower at each *E*_FE_ and requires larger electric field at each *t*_pw_ than program operation for the thin HZO capacitors, which indicates the asymmetry in polarization switching.

To investigate the thickness dependence of the switching characteristics, the nucleation-limited-switching (NLS) model [22, 23] was used for modeling. The NLS model describes macroscopic polarization switching as an ensemble of independent switching in nucleated domains. A ferroelectric-HfO_2_ unit cell contains movable and non-movable oxygen planes alternately. Unlike perovskite ferroelectric, ionic dipoles are weakly coupled with each other. Polarization switching in poly-crystalline HfO_2_-based ferroelectric thin film can be dominated by nucleation growth rather than domain wall propagation. Therefore, the NLS model is commonly used. In the field-dependent NLS model, the cumulative probability of the switching time is described as,

1 $$P(t|\tau ,\beta ) = 1 - \exp \left[ { - \left( {\frac{t}{\tau }} \right)^{\beta } } \right]$$

where β is a stretched exponential parameter and τ is a switching time constant which depends on the applied electric field *E*_FE_ as,

2 $$\tau (E_{a} ,E_{FE} ) = \tau_{\min } \exp \left[ {\left( {\frac{{E_{a} }}{{E_{FE} }}} \right)^{\alpha } } \right]$$

where *τ*_min_ is a coefficient of τ and α is an empirical exponential parameter. *E*_a_ is an activation field which can have a distribution representing the variability of grains and domains in a film. An effective activation field is expressed as *E*_a_’ = *E*_a_/*η*, where *η* is a random variable that follows statistical distribution. The total switching probability is then calculated by,

3 $$P(E_{{\text{FE}}} ,t) = \int_{0}^{\infty } {P(t|{\uptau }({\upeta }^{ - 1} E_{a} } ,E_{{\text{FE}}} ),{\upbeta })f({\upeta })d{\upeta }$$

where *E*_a_, *τ*_min_, α, β and *f*(*η*) are fitting dynamic parameters. Measured normalized switched polarization can be fitted by this model and the dynamic parameters can be extracted.

**Fig. 6:**
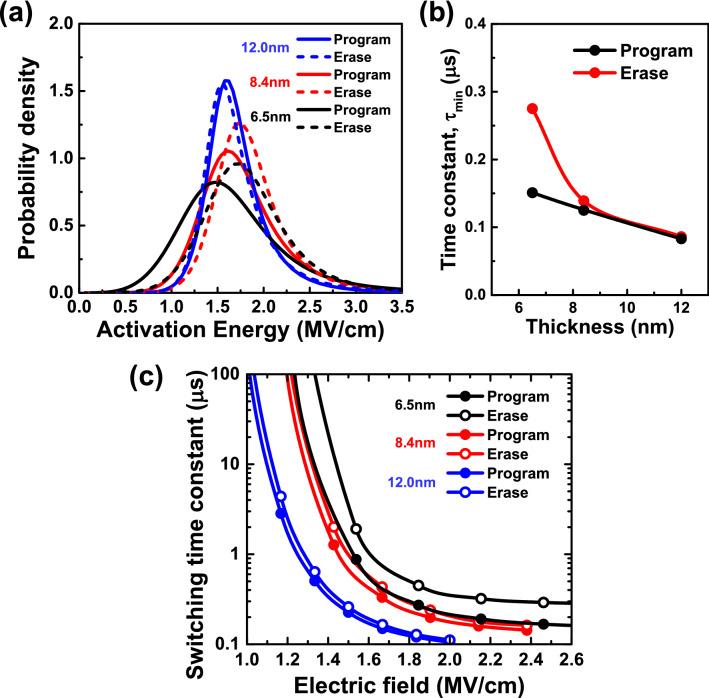
Extracted (**a**) *E*_a_’ distribution and (**b**) *τ*_min_ for program and erase operation, for different HZO thickness, (**c**) calculated average time constants < *τ* > versus *E*_FE_ for program and erase operation, for different HZO thickness

The extracted dynamic parameteres *E*_a_’ and τ_min_ are shown in Fig. 6 for all thicknesses and program and erase operation. The thick HZO capacitor shows a sharp distribution of *E*_a_’, while the thin HZO capacitor shows a broad distribution of *E*_a_’. The long tail in the *E*_a_’ distribution of the thin HZO capacitor means large electric field is required for completely switching polarization, which leads to slow switching in the thin HZO capacitor. In addition, *E*_a_’ is distributed in larger value for erase operation than for program operation, which leads to slow switching in erase operation. *τ*_min_ is less sensitive to HZO thickness for the thick HZO capacitor and almost the same for program and erase operation. For the thin HZO capacitor, however, *τ*_min_ becomes larger in erase operation than in program operation. Assymetry in switching kinetics becomes distinct for the thin HZO capacitors, which will be discussed shortly. By using the extracted parameters, average switching time constant < *τ* > can be calculated by integrating *τ* with the distribution *f*(*η*), as shown in Fig. 6c. < *τ* > represents the thickness dependence of switching speed and its asymmetry.

**Fig. 7:**
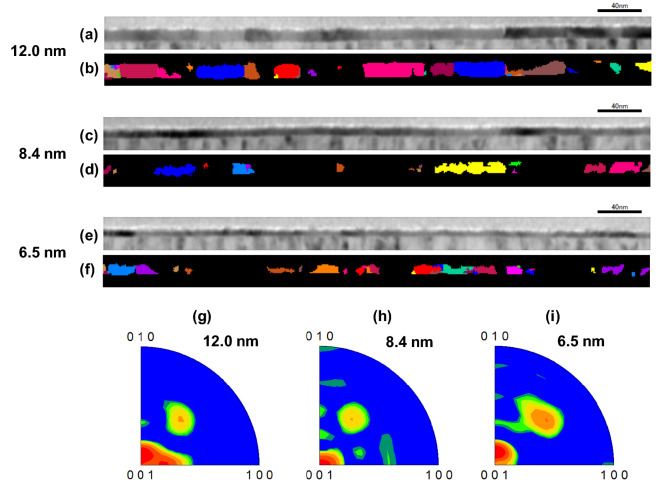
Cross-sectional bright-field TEM images and f-phase grain maps for the (**a**, **b**) 12.0 nm, (**c**, **d**) 8.4 nm, (**e**, **f**) 6.5 nm-thick HZO capacitors. Inverse pole maps of orthorhombic grains for the (**g**) 12.0 nm, (**h**) 8.4 nm, (**i**) 6.5 nm-thick HZO capacitors with respect to the (001) orientation. The color represents the population of the orientations (red: highest, green: medium, blue: lowest)

To investigate the material property that causes the thickness dependence of the switching kinetics, cross-sectional TEM images were taken for all thicknesses as shown in Fig. 7a, c, e. The samples received 10^3^ electrical cycling for wake-up of the HZO capacitors and the top electrodes were removed before analysis. Then, electron diffraction mapping was used to identify the grain size and grain distribution of the f-phase in the TEM images as shown in Fig. 7b, d, f. The HZO layers are poly-crystalline and the computed average grain size is 10.1 nm, 8.8 nm, and 7.3 nm for 12.0 nm-, 8.4 nm, and 6.5 nm-thick HZO layers, respectively. Large grain formation is suppressed in the thin HZO capacitors [12, 24]. There exist more grain boundaries which can be electrically charged and pin the polarization [25]. Thus, polarization switching is slower and requires larger electric field in the thinner HZO capacitors. Figure 7g, h, i show inverse pole maps of the f-phase grains, which illustrates the distribution of crystalline axis of the f-phase grains. For thicker HZO, out-of-plane (001) orientation is dominant, however, for thinner HZO, other orientation such as (111) is also prominent. This large variability in ferroelectric grains can be one of the reasons for the wide distribution of *E*_a_’ in Fig. 6a. The asymmetric switching kinetics for program and erase operation in the thin HZO capacitor can be attributed to the structural asymmetry of the MFM capacitor. Oxygen atoms are exchanged at the interface during the thermal process, and thus, a TiON interfacial layer and oxygen vacancy are formed [26]. The bottom HZO/TiN interface receives more thermal budget than the top TiN/HZO interface, including ALD deposition process. Therefore, a thicker TiON layer and more oxygen vacancies are formed at the bottom interface [27–29]. In erase operation, the initialization pulse induces electron trapping at the bottom HZO/TiN interface in polarization switching. Then, the erase pulse needs to detrap the electrons through the interfacial layer in reverse polarization switching. Thus, polarization switching is slower and requires larger electric field in erase operation. The asymmetry is more prominent in the thin HZO capacitor because more voltage is applied at the interface region at a given applied electric field. This result indicates that very thin HZO film may not gain as much scaling benefit as predicted by linearly scaling thin HZO film characteristics but require process engineering and optimization.

### The polar-axis alignment of ferroelectric grains with electric field

**Fig. 8:**
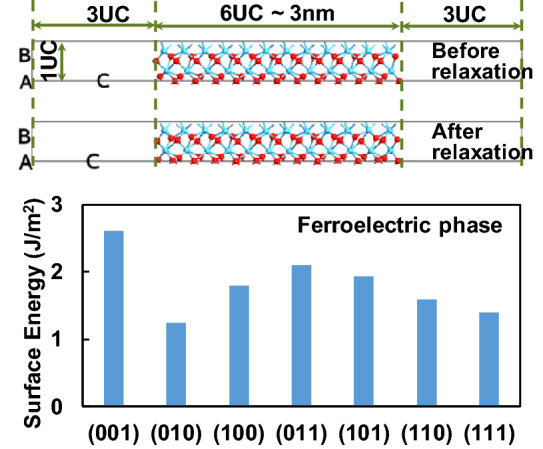
Calculated surface energies of ferroelectric-HfO_2_ slabs with different orientation

We start from identifying the preferable crystalline orientation of the f-phase grain by using ab-initio calculation [17]. Figure 8 shows the calculated surface energy of the f-phase for different primary surface orientation. It turns out that out-of-plane polar (001) orientation has the largest surface energy, while in-plane polar (010) orientation has the smallest surface energy. In-plane polar (100) orientation has medium surface energy. Therefore, the in-plane polar orientation is naturally stable in the HfO_2_-based ferroelectric thin film.

**Fig. 9:**
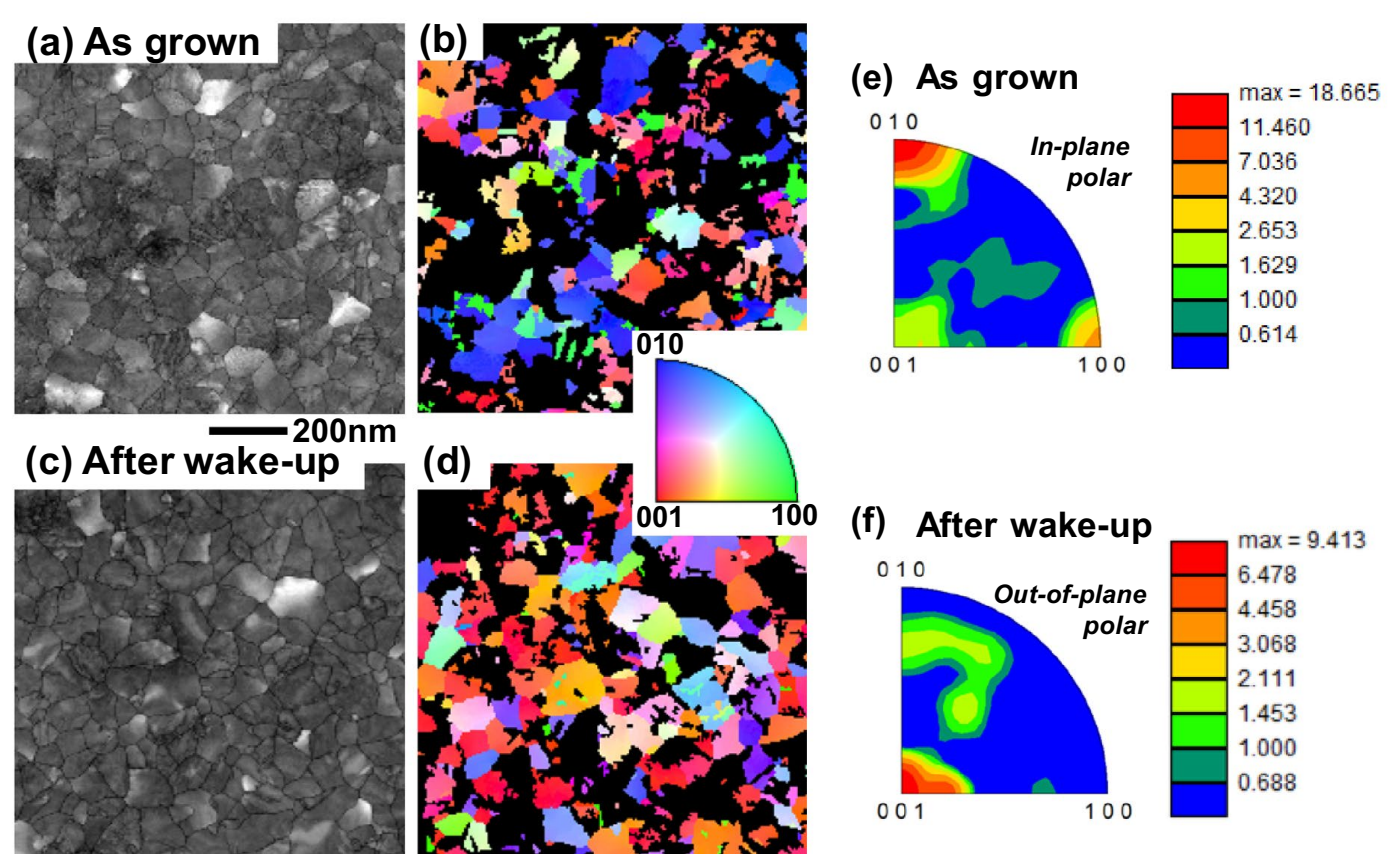
Plan-view TEM images and crystal-orientation color-maps of HZO films (**a**, **b**) without and (**c**, **d**) with wake-up operation. Inverse polar maps (**e**) without and (**f**) with wake-up operation by 10^3^ electrical cycling

To experimentally identify the orientation of the f-phase grains, two MFM capacitors were fabricated with 10 nm-thick HZO layers. After crystallization anneal, 10^3^ electrical cycling was applied on one sample for wake-up and electrical test, while the other sample was kept as grown. Plan-view TEM images were taken for both samples after stripping the top TiN electrode as shown in Fig. 9a, c. Then, electron diffraction mapping was carried out on the same samples. Figure 9b, d show individual f-phase grains across the films (the black color region represents other phase grains). Inverse pole maps are generated based on Fig. 9b, d in Fig. 9e, f. In the as-grown sample, in-plane polar (010) orientation is dominant and in-plane polar (100) orientation is also highly populated, while out-of-plane polar (001) orientation slightly exists. This result is consistent with the previous theoretical prediction in Fig. 8. On the other hand, in the sample with wake-up cycling, out-of-plane polar (001) orientation becomes dominant, while in-plane polar (010) and (100) orientation are not populated. This indicates that the in-plane polar (010) and (100) orientation grains transit to the out-of-plane polar (001) orientation grains under the electric field.

**Fig. 10:**
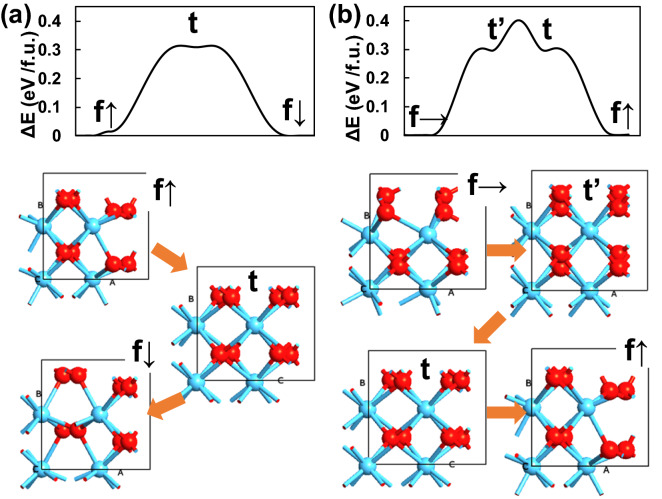
Calculated kinetic pathway of (**a**) up/down polarization switching and (**b**) in-plane/out-of-plane polarization transition via t-phases as intermediate steps

To verify this polar-axis transition of the f-phase grains, ab-initio calculation was used to identify the kinetic pathway of the transition. Total energy was calculated along the movement of oxygen atoms. Figure 10a shows the calculated kinetic pathway of nominal polarization switching along (001) direction. Both up and down polarized states have the lowest energy. The transition occurs through the metastable t-phase (denoted as t) along the movement of oxygen atoms in the out-of-plane polar axis. Figure 10b shows the calculated kinetic pathway of the transition from in-plane polar (010) orientation to out-of-plane polar (001) orientation. Both polar states are the stable states in the beginning and end. Starting from the f-phase with in-plane polar (010) orientation, a t-phase (denoted as t’) appears along the movement of oxygen atom in the in-plane polar axis. This t-phase has similar energy as the t-phase in Fig. 10a. Then, there exists a potential barrier which separate the two t-phases, t’ and t [30]. This potential barrier is not as high as the potential barrier for up and down polarization switching and can be overcome by the electric field. By overcoming the potential barrier, the t-phase (denoted as t) appears. Then, the electric field further moves oxygen atoms in the out-of-plane polar axis, and finally the f-phase with out-of-plane polar (001) orientation is realized. Therefore, even if the as-grown HZO film contains in-plane polar orientation grains, the polar-axis can transit from in-plane to out-of-plane by applying electric field. Large ferroelectricity can be obtained in poly-crystalline HZO films. This feature of HfO_2_-based ferroelectric is highly beneficial for 3D structure ferroelectric memory which requires highly polarized grains with respect to any type of surface in 3D structure.

## Conclusions

We studied the thickness dependence of the polarization switching kinetics in HfO_2_-based ferroelectric. Although low-frequency static polarization is comparable for different thickness, dynamic polarization switching speed is different and the thinner HZO capacitors show slower switching speed and require larger electric field for switching. Based on the analysis using the NLS model and physical characterization, thinner HZO contains smaller grains with orientation non-uniformity and more grain boundaries than thicker HZO, which can impede macroscopic polarization switching. Large size grain formation and/or passivating grain boundaries are important for fast polarization switching operation. Asymmetry in switching characteristics for program and erase operation can be due to the different interface property between top and bottom interface. Process engineering and optimization will be required to gain the benefit of thickness scaling.

We also studied the polar-axis alignment of HfO_2_-based ferroelectric poly-crystalline thin films. In-plane polar orientation is stable in as-grown HZO, which is theoretically and experimentally observed. However, by applying electric field, out-of-plane polarization can be dominant which indicates the transition from in-plane polar to out-of-plane polar orientation in the f-phase grains. This is confirmed by calculating kinetic pathway using ab-initio calculation. The feature of the field-induced polar-axis alignment in HfO_2_-based ferroelectric is encouraging for 3D ferroelectric devices.

## Data Availability

The data that support the findings of this study are available from the corresponding author upon reasonable request.
